# Reconstructing the cytokine view for the multi-view prediction of COVID-19 mortality

**DOI:** 10.1186/s12879-023-08291-z

**Published:** 2023-09-21

**Authors:** Yueying Wang, Zhao Wang, Yaqing Liu, Qiong Yu, Yujia Liu, Changfan Luo, Siyang Wang, Hongmei Liu, Mingyou Liu, Gongyou Zhang, Yusi Fan, Kewei Li, Lan Huang, Meiyu Duan, Fengfeng Zhou

**Affiliations:** 1 College of Computer Science and Technology, Jilin University, 130012 Changchun, China; 2 School of Biology and Engineering, Guizhou Medical University, 550025 Guiyang, Guizhou China; 3 Key Laboratory of Symbolic Computation and Knowledge Engineering of Ministry of Education, Jilin University, 130012 Changchun, China; 4 Department of Epidemiology and Biostatistics, School of Public Health, Jilin University, 130021 Changchun, Jilin Province China; 5 College of Software, Jilin University, 130012 Changchun, China; 6 Engineering Research Center of Medical Biotechnology, Guizhou Medical University, 550025 Guiyang, Guizhou China

**Keywords:** Model-adjusted cytokine, Mortality prediction, COVID-19, Cytokine prediction, Complete blood counts

## Abstract

**Background:**

Coronavirus disease 2019 (COVID-19) is a rapidly developing and sometimes lethal pulmonary disease. Accurately predicting COVID-19 mortality will facilitate optimal patient treatment and medical resource deployment, but the clinical practice still needs to address it. Both complete blood counts and cytokine levels were observed to be modified by COVID-19 infection. This study aimed to use inexpensive and easily accessible complete blood counts to build an accurate COVID-19 mortality prediction model. The cytokine fluctuations reflect the inflammatory storm induced by COVID-19, but their levels are not as commonly accessible as complete blood counts. Therefore, this study explored the possibility of predicting cytokine levels based on complete blood counts.

**Methods:**

We used complete blood counts to predict cytokine levels. The predictive model includes an autoencoder, principal component analysis, and linear regression models. We used classifiers such as support vector machine and feature selection models such as adaptive boost to predict the mortality of COVID-19 patients.

**Results:**

Complete blood counts and original cytokine levels reached the COVID-19 mortality classification area under the curve (AUC) values of 0.9678 and 0.9111, respectively, and the cytokine levels predicted by the feature set alone reached the classification AUC value of 0.9844. The predicted cytokine levels were more significantly associated with COVID-19 mortality than the original values.

**Conclusions:**

Integrating the predicted cytokine levels and complete blood counts improved a COVID-19 mortality prediction model using complete blood counts only. Both the cytokine level prediction models and the COVID-19 mortality prediction models are publicly available at http://www.healthinformaticslab.org/supp/resources.php.

**Supplementary Information:**

The online version contains supplementary material available at 10.1186/s12879-023-08291-z.

## Introduction

The new coronavirus pneumonia (COVID-19) is caused by the severe acute respiratory syndrome coronavirus 2 (SARS-CoV-2) [1]. The disease has spread across 222 countries and caused more than six million deaths [2].

Because of the severe and rapidly developing symptoms of this disease, multiple studies have investigated prognosis prediction models for COVID-19 patients. Iwendi C et al. collected geographical information, travel histories, symptoms, and demographic data for the mortality prediction of COVID-19 patients [3]. The AdaBoost strategy boosted a random forest classification model and trained it over the collected data to achieve a prediction accuracy of 94%. Sankaranarayanan S et al. conducted a retrospective investigation of the mortality prediction using the COVID-19 patients recorded in the Mayo Clinic system [4]. A modified gated recurrent unit with trainable decay weight (GRU-D) network was established using the electronic health records of their cohort shortly after their confirmation of SARS-CoV-2 infections. Finally, Ko H et al. fully used the prediction capabilities of deep neural networks and random forest (RF) models to build an ensemble model and achieved a COVID-19 mortality prediction accuracy of 92% [5].

Cytokine storm is one of the major factors leading to the exacerbation and even death of COVID-19 patients [6]. Children with COVID-19 pneumonia showed higher levels of serum interleukin-6 (IL-6), interleukin-10 (IL-10), and tumor necrosis factors-α (TNF-α) than children without pneumonia [7]. The cytokine concentration was also observed at admission in deceased COVID-19 patients [8]. For example, the interleukin-8 (IL-8) levels were connected with the in-hospital deaths of severe/critical COVID-19 patients [9]. Multiple studies have shown the powers of these cytokines in predicting the severity of COVID-19 patients so that clinical practitioners and medical resources may be optimally deployed [10].

This study provided an *in silico* solution for detecting cytokine levels using complete blood counts [11] of COVID-19 patients. The detection of cytokine levels relied on specialized medical devices such as the optical microfiber reader and nanoplasmonic immunoassay [12, 13], which are not accessible in economically underdeveloped areas. This study conducted a comprehensive evaluation of the quantitative correlations between cytokines and complete blood counts. The autoencoder (AE) network was used to filter the noisy background information of the complete blood count data. The decoded complete blood counts were then enriched using principal component analysis (PCA). A regression model based on the enriched principal component (PC) features was trained for the level of each cytokine. The comparative experiments showed that some cytokines’ *in silico* estimated levels showed even more significant associations with COVID-19 mortality than their original levels.

## Materials and methods

### Datasets

One of the largest COVID-19 datasets was released from the Tongji Hospital, and detailed information on the recruitment and inclusion/exclusion procedures has been described in [14]. The Ethics Committee of Tongji Hospital approved the study. The data were collected from the patients in Wuhan, China, from January 10 to February 18, 2020. The study excluded the data of pregnant or lactating women and patients under 18 years of age, and the data records with a completeness rate of less than 80% were also excluded. Follow-up time is defined as the duration from admission to death or discharge.

This study used two feature views of the samples, i.e., complete blood counts and cytokine levels. The samples without both feature views were excluded from this study. Only the first complete blood count data were collected for investigations. Patients with the first tests of complete blood counts (dataset A) were split into two datasets, i.e., patients with inflammatory cytokine levels (A1) and without cytokine levels (A2). Dataset A1 was then randomly split into 70% training (A1-Training) and 30% testing samples (A1-Testing). The samples in each dataset are summarized in Table 1. There were two groups of samples, i.e., Deceased and Survived. Datasets A1 and A2 used the study’s first complete blood count data. The baseline characteristics of dataset A are summarized in Additional File 1: Table S1. Only the complete blood counts statistically significantly associated with the mortality in the samples, with both complete blood counts and cytokine levels were kept for further analysis.

**Table 1:** Summary of the samples in each dataset

	A1	A1-Training	A1-Testing	A2
**Deceased**	82	57	25	68
**Survived**	121	85	36	68

This study investigated the mortality prediction of COVID-19 patients, i.e., a deceased patient was a positive sample, and a patient who survived was regarded as a negative sample.

### Feature selection and classification algorithms

Three feature selection algorithms were used to find the best features for the prediction models. First, the features were weighted and ranked in descending order of their importance in the trained models using support vector machine (SVM), adaptive boost (AdaBoost), and RF [15, 16]. Then the incremental feature selection (IFS) strategy [17] was used to find the best subset of features for predicting the mortality of COVID-19 patients.

The chosen features were evaluated for the prediction performances using five classifiers, i.e., SVM, RF, K-nearest neighbor (KNN), decision tree (CART), and the Bernoulli naïve Bayes (NB). SVM learned the maximized interval between the two classes of samples and showed good classification performances on small datasets [18]. CART constructed a decision tree from the root node by iteratively dividing the dataset into relatively homogeneous subsets using eigenvalues [19]. RF was an ensemble learning method based on multiple baseline decision tree models for classification or regression [20]. The basic idea of KNN was to assign a test sample to the class where most of this test sample’s KNNs belong based on the pre-defined similarity measure [21]. Finally, NB assumed the inter-feature independence and applied the Bayesian theorem to calculate the probability of a test sample belonging to each class [22].

### Feature engineering

**Fig. 1:**
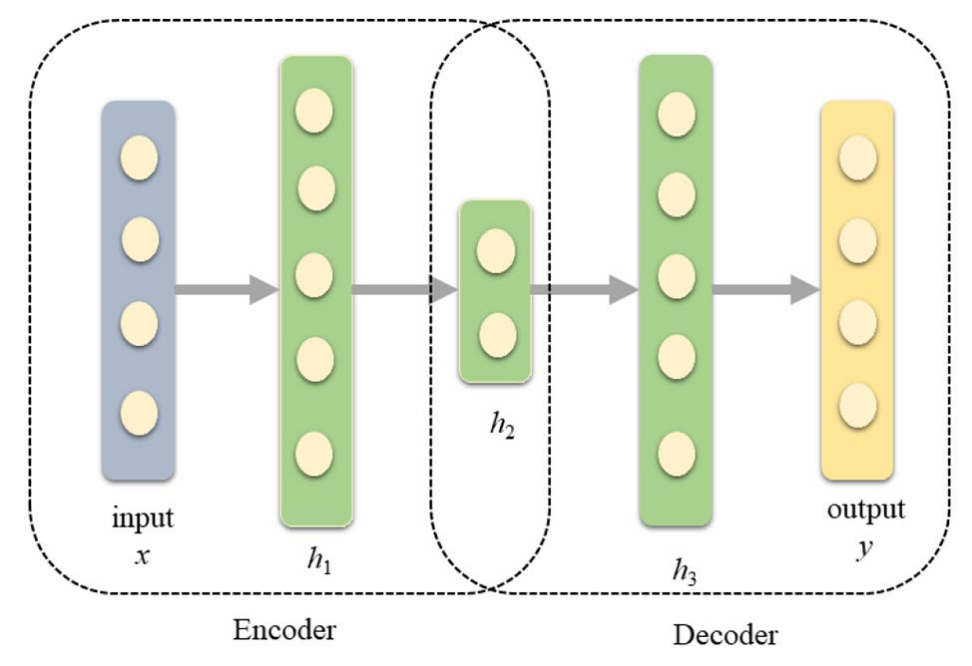
The architecture of the autoencoder

AE consists of two sub-networks, encoder and decoder, as illustrated in Fig. 1. The encoder sub-network maps the input high-dimensional data *x* into one or several hidden layers. The decoder sub-network reconstructs the output data *y* from the encoded information to minimize the difference between *y* and *x* results. AE tends to retrain only features with high variabilities [19].

PCA was mathematically defined to achieve orthogonal linearity and was fully invertible. PCA has been widely used for various purposes, including dimensionality reduction, noise suppression, visualization, and data compression [23–25]. This study applied PCA to the trained AE network’s hidden and output layers. The calculated PCs served as the engineered features of a regression model to predict cytokine levels. These regression algorithms were used, including gradient boosting regressor (GBR), random forest regressor (RFR), support vector regression (SVR), and linear regression (LR) [26–29].

### Statistical analysis and performance evaluation metrics

The area evaluated a binary classification model under the receiver operating characteristic (ROC) curve (AUC), accuracy (Acc), sensitivity (Sn), and specificity (Sp) [30]. Sn and Sp measured the percentages of the correctly predicted positive and negative samples, respectively [31]. The prediction accuracy (Acc) was defined as the proportion between the number of correctly classified samples and that of all the samples [32]. AUC was an assessment metric between Sn and Sp and served as a parameter-independent metric for a binary classification model [33].

All the experiments were implemented using Python version 3.8.8, sci-kit-learn version 1.0.2, and PyTorch version 1.10.1.

### Workflow of this study

**Fig. 2:**
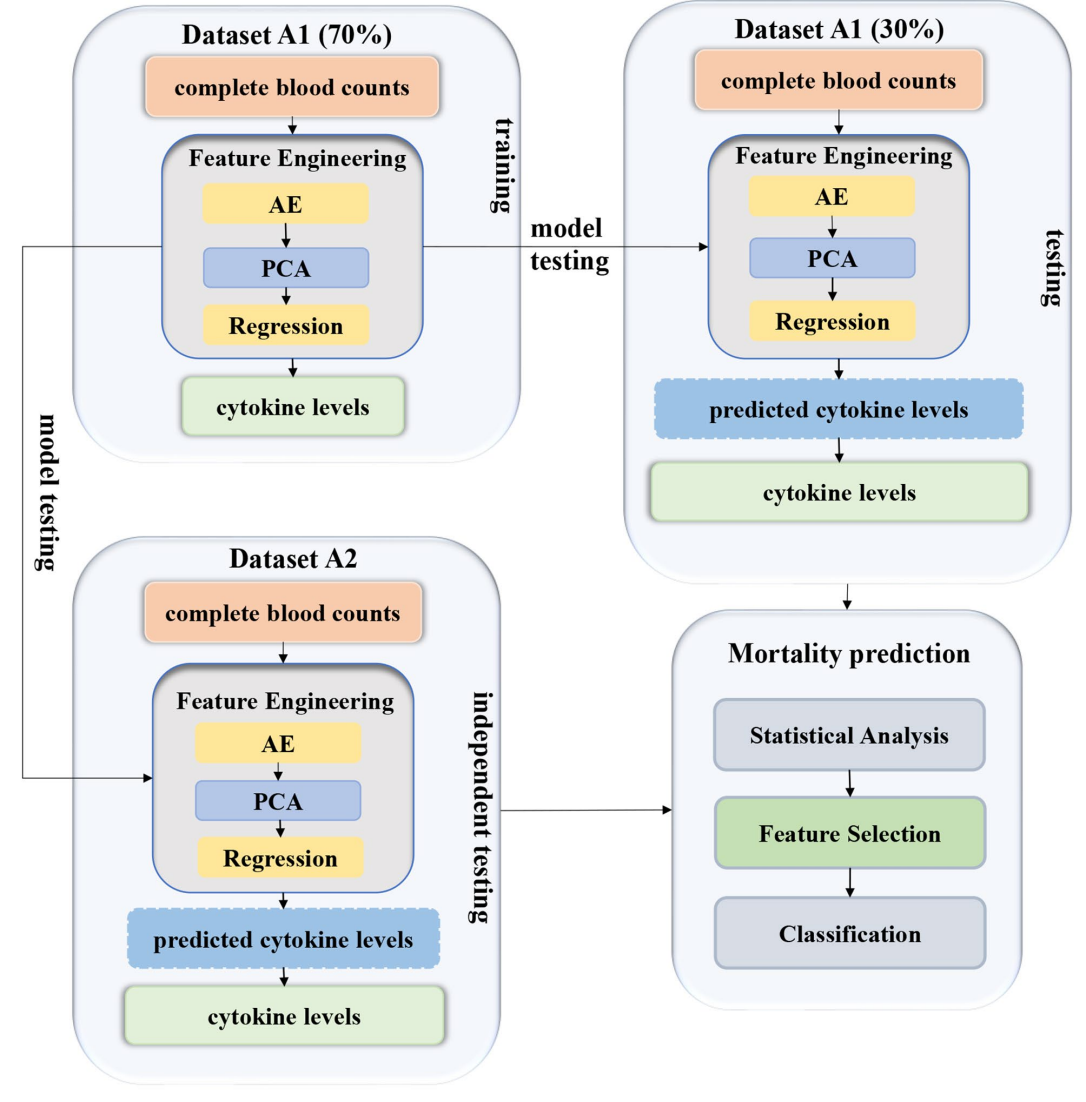
Experimental workflow of dataset A in this study. A similar workflow was performed on dataset B

This study conducted the experiments as illustrated in Fig. 2. First, a regression model for the cytokine levels was optimized based on the complete blood counts in the A1-Training and was tested in the dataset A1-Testing. This study used 70% of the samples in dataset A1 as the training set and then trained the prediction model for the cytokine levels based on the information of the complete blood count. Then the predicted cytokine levels and the real complete blood counts were used to predict the prognosis of the disease in the remaining 30% of dataset A1. Finally, the samples of dataset A2 did not have cytokine levels and were used as the independent testing dataset to validate the model externally.

## Results

### Both complete blood counts and cytokine levels are important for the COVID-19 mortality prediction task

**Fig. 3:**
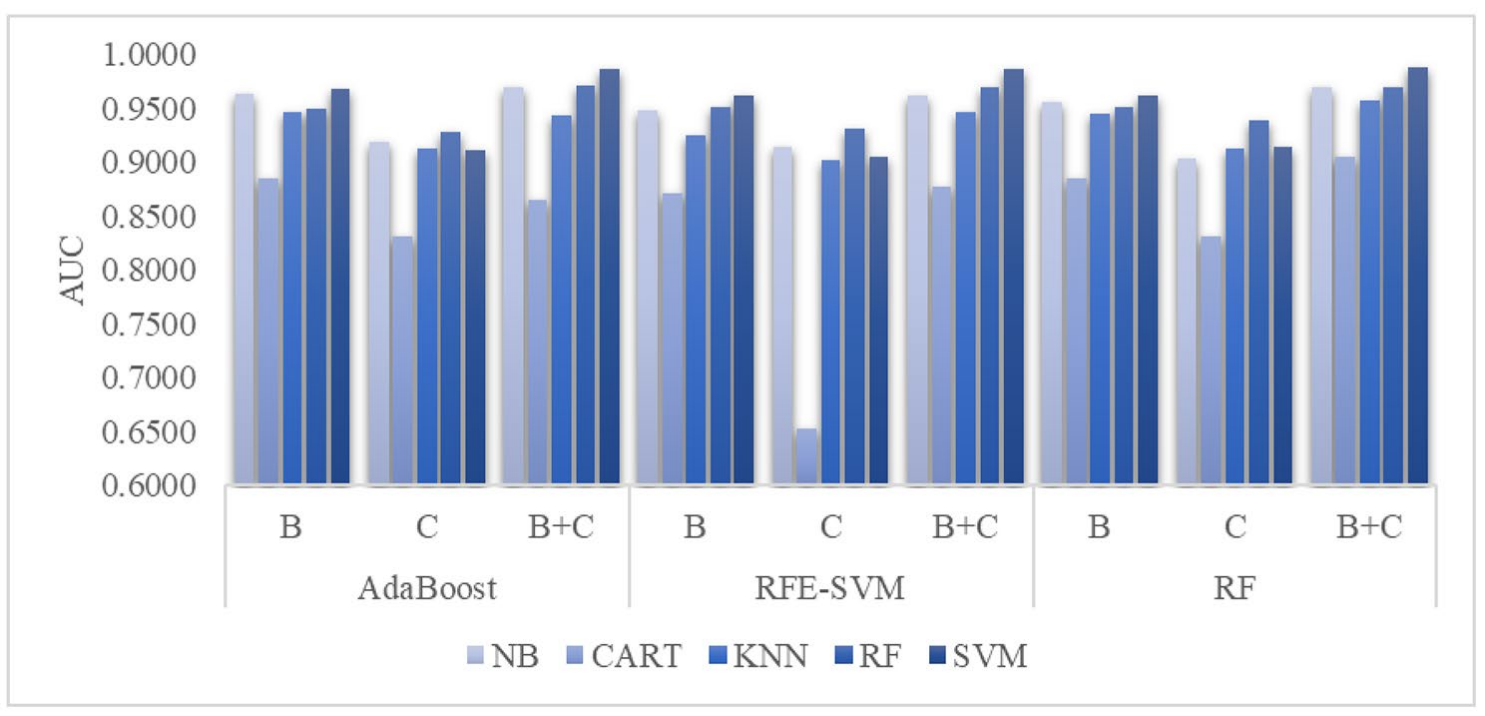
The mortality prediction of COVID-19 patients. This experiment used three feature selection algorithms, i.e., AdaBoost, RFE-SVM, and RF. The classification models were trained using five classifiers, i.e., NB, CART, KNN, RF, and SVM. The data series B, C, and B + C represent the models using complete blood counts only, cytokine levels only, and both datasets, respectively. Each model was trained using the dataset A1-Training and tested on the dataset A1-Testing.

We evaluated complete blood counts and cytokine levels on the COVID-19 mortality prediction task, as shown in Fig. 3-the 203 samples in dataset A1 were used in this experiment. Three feature sets were evaluated, i.e., complete blood count features only (B), cytokine levels only (C), and both feature sets (B + C). Three feature selection algorithms were used to screen for the mortality-associated features. The ROC curve was defined as the relationship between the metrics Sn and its 1-Sp on the testing dataset. The metric AUC was the area under the ROC curve, and it is a popularly used metric to measure the overall performance of a binary classifier [34–36]. A larger AUC value suggests a better classification model. Therefore, we used AUC to evaluate the classification performance of the five classifiers.

The classifier SVM achieved the best AUC values on the two datasets, B and B + C, when collaborating with all three feature selection algorithms, as shown in Fig. 3. In addition, the best overall AUC = 0.9878 for the COVID-19 mortality prediction task was achieved by SVM using the RF-selected features.

The classifier RF achieved the best AUC values, namely 0.9272, 0.9306, and 0.9383, using the cytokine levels selected by the three feature selection algorithms, AdaBoost, RFE-SVM, and RF, respectively. However, slightly decreased AUC values of 0.9111, 0.9056, and 0.9133 were achieved by the classifier SVM using the cytokine levels selected by the three feature selection algorithms (AdaBoost, RFE-SVM, and RF, respectively).

It was also interesting to observe that the complete blood count and cytokine level contributed useful information for the COVID-19 mortality prediction task. Their combination achieved even better prediction performances. The cytokine levels alone facilitated the mortality prediction, with an AUC as large as 0.9383. The best mortality prediction (AUC = 0.9678) based on the complete blood count was achieved by the classifier SVM using the AdaBoost-selected features. If we used both complete blood counts and cytokine levels, the overall best prediction (AUC = 0.9878) was achieved by the classifier SVM.

This study hypothesized that we could use the cytokine level predicted using the complete blood count for the COVID-19 mortality prediction task if the cytokine level detection devices were unavailable. Thus, we used the combination of the feature selection algorithm AdaBoost and the classifier SVM to achieve the best AUC value for the complete blood count.

### Choice of the regression model in feature construction

**Fig. 4:**
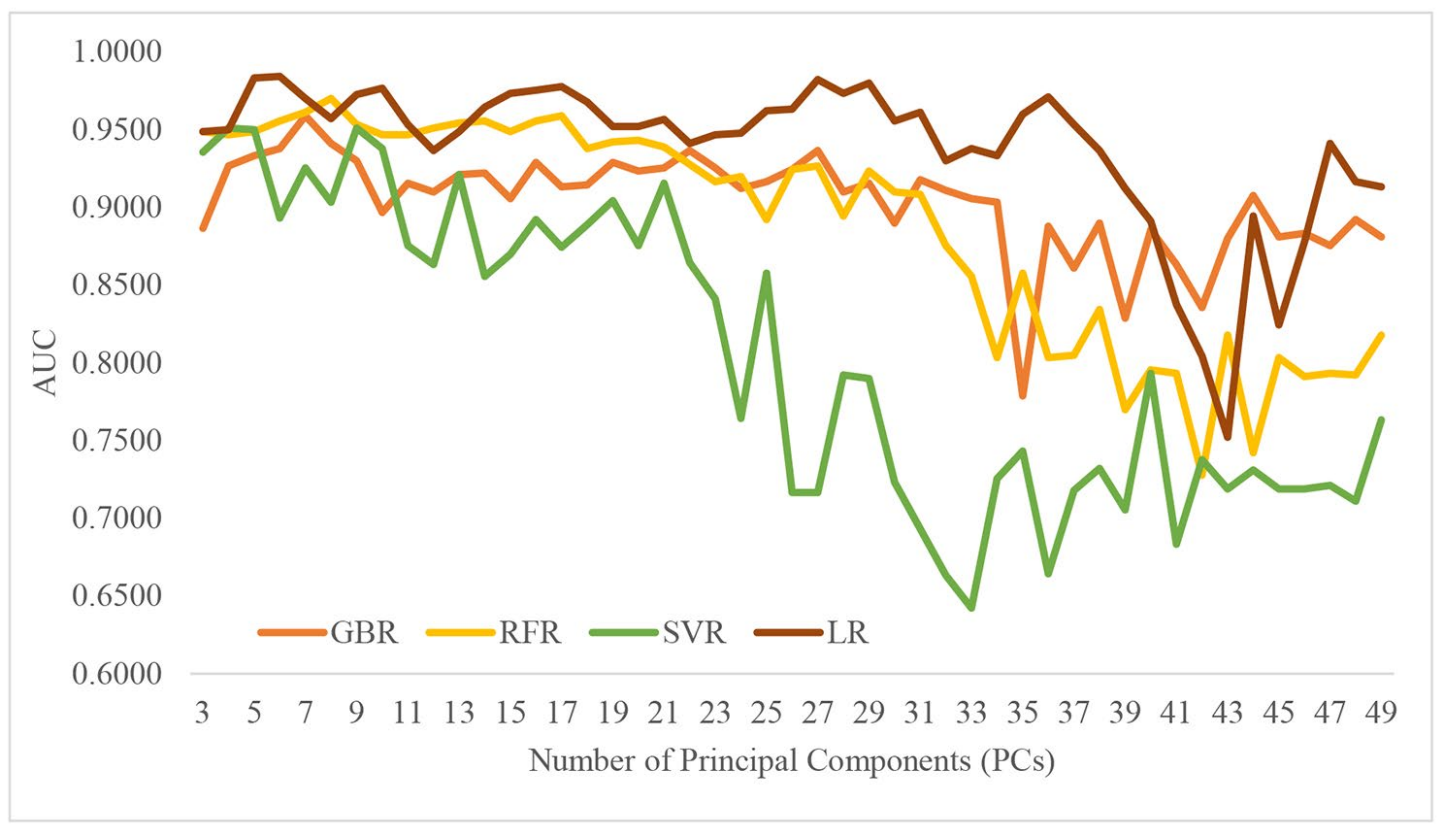
Mortality classifications are based on the cytokine levels estimated by the regression models. The training process of the cytokine level regression models was conducted on the dataset A1-Training. First, the AE network filtered the complete blood counts and then enriched them with PCA. Next, different PCs were loaded to train the regression model to each cytokine level. Finally, the mortality prediction was conducted on the dataset A1-Testing.

Figure 4 illustrates that all four regression models generated the predicted cytokine levels outperforming the original cytokine levels on the mortality classification performances. PCA is a linear dimensionality reduction algorithm that uses variance to measure the difference of data and projects high-dimensional data into a low-dimensional representation space. To reduce the dimension of the input features of the regression, we used PCA to reduce the impact of data noise for the prediction model. We observed that the model’s classification performance differed when the number of PCs was selected (Fig. 4). Therefore, we chose the number of the top-ranked PCs with the best classification metric AUC value for the regression model of prognosis prediction.

The regression algorithm LR delivered the overall best mortality classification (AUC = 0.9844) using six PCs. RFR used eight PCs to estimate cytokine levels and was ranked as the second-best regression algorithm (AUC = 0.9700). The cytokine levels estimated by GBR and SVR achieved the best mortality classification (AUC values of 0.9589 and 0.9511, respectively). The original cytokine levels only achieved an AUC value of 0.9383 for the prediction of COVID-19 mortality. This study used the levels of six cytokines, i.e., interleukin-2 receptor (IL-2R), interleukin-8 (IL-8), interleukin-10 (IL-10), tumor necrosis factor-α (TNF-α), interleukin-1β (IL-1β), and interleukin-6 (IL-6). The final best mortality prediction model achieved an AUC value of 0.9844 using five of the six LR-estimated cytokine levels, and the level of IL-6 was excluded from the model.

**Fig. 5:**
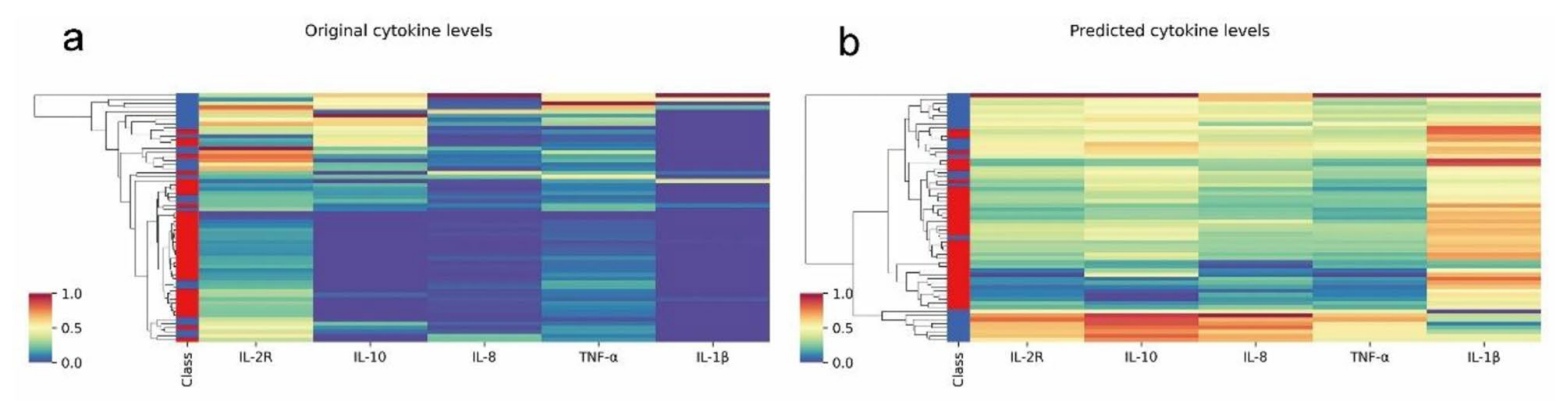
Visualization of the original and predicted levels of the five cytokines. The heatmaps were generated for the (**a**) original and (**b**) predicted levels of the five cytokines after the standard scaling. The samples were hierarchically clustered using the respective cytokine levels. The “Deceased” and “Survived” samples were colored red and blue, respectively

A further evaluation of the regression performances of the five cytokine levels was conducted for the best regressor LR, as shown in Fig. 5. The heatmap (Fig. 5a) shows that the original cytokine levels had skewed distributions toward 0, while the predicted cytokine levels had smoothed distributions (Fig. 5b). In addition, the predicted cytokine levels showed a slightly better discrimination power for COVID-19 mortality by the simple hierarchical clustering in the heatmaps. This might be due to the radical fluctuations of the inflammatory cytokine levels in COVID-19 patients. In addition, the model-corrected cytokine levels showed much stronger associations with COVID-19 mortality.

The following sections used LR as the regressor for the six cytokine levels, and the mortality prediction model used the levels of the five cytokines, i.e., IL-1β, IL-2R, IL-8, IL-10, and TNF-α.

### Statistical analysis of the original and predicted cytokine levels

**Fig. 6:**
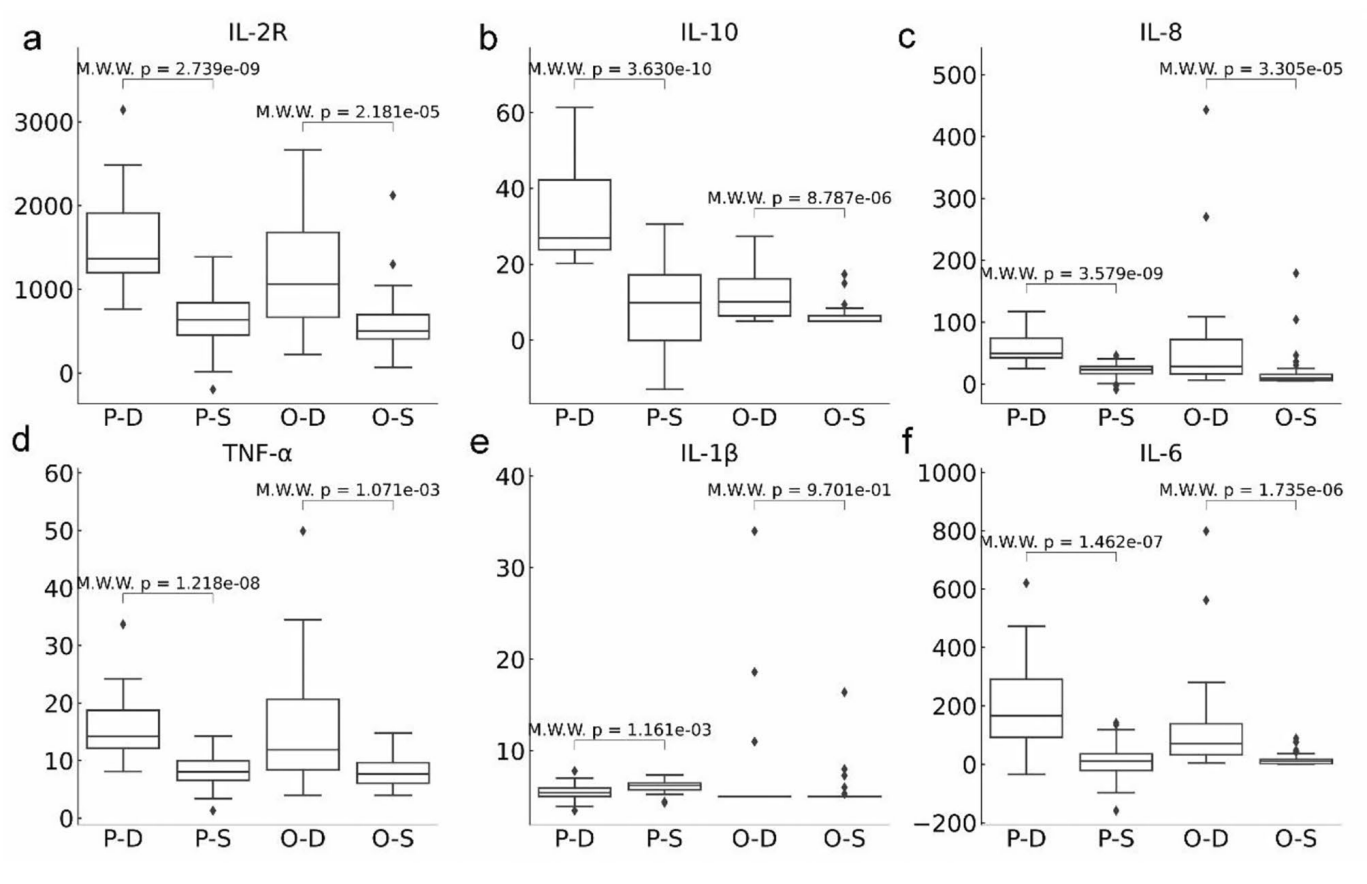
Statistical analysis of the original and predicted cytokine levels on the dataset A1-Testing. The statistical significance *p*-value was calculated using the Mann-Whitney Wilcoxon (abbreviated as M.W.W.) test. The P-D and P-S groups represented the predicted cytokine levels in the deceased and survived COVID-19 patients, respectively. In contrast, the groups O-D and O-S were the original cytokine levels of the deceased and survived patients, respectively. IL-2R: Interleukin-2 receptor, IL-8: Interleukin-8, IL-10: Interleukin-10, TNF-α: Tumor necrosis factor-α, IL-1β: Interleukin-1β, and IL-6: Interleukin-6

We evaluated the statistical associations of each cytokine’s original and prediction levels with COVID-19 mortality, as shown in Fig. 6. The Mann-Whitney Wilcoxon test was used because there were a limited number of samples, and most of the cytokine levels did not strictly follow the normal distributions [37]. The original levels of five cytokines were statistically significantly associated with COVID-19 mortality, i.e., IL-6 (*p* < 0.001), IL-2R (*p* < 0.001), IL-8 (*p* < 0.001), IL-10 (*p* < 0.001), and TNF-α (*p* < 0.05). The original level of IL-1β showed no statistical significance (*p* = 0.9701) with COVID-19 mortality. While the predicted levels of all six cytokines showed significant associations, their associations with COVID-19 mortality were more significant than their original levels.

The statistical analysis suggested that the predicted cytokine levels captured the hidden inflammatory parts of the cytokine levels with significant associations with COVID-19 mortality, while the original cytokine levels may also always accomplish, e.g., IL-1β.

### Contributions of the predicted cytokine levels

**Fig. 7:**
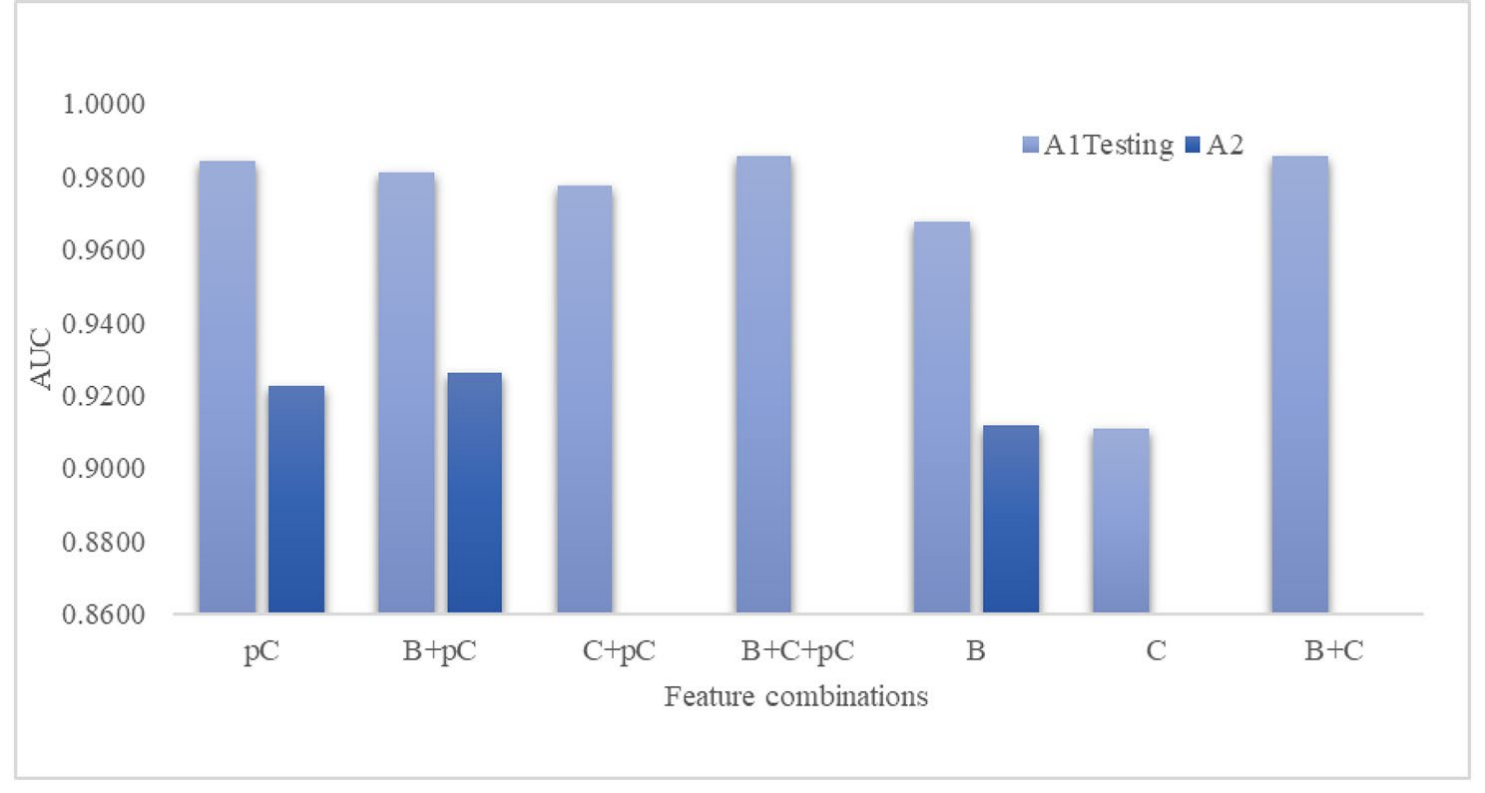
The COVID-19 mortality classifications use different feature combinations. The complete blood counts and cytokine levels were denoted as feature sets B and C, respectively. The predicted cytokine levels were denoted as the feature set pC. The cytokine level prediction models were trained using the dataset A1-Training. The evaluation was conducted using the dataset A1-Testing and the independent test dataset A2. Because dataset A2 lacked the original cytokine levels, some feature combinations did not show the COVID-19 mortality classification AUC values. The vertical axis displays the classification AUC value

We further evaluated how the predicted cytokine levels contributed to the COVID-19 mortality classification task, as shown in Fig. 7. First, the classification models using only complete blood counts (B) or original cytokine levels (C) achieved the AUC values of 0.9678 and 0.9111, suggesting that both complete blood counts and cytokine levels were important to predict the mortality of COVID-19 patient. Interestingly, the classification models were improved by adding the feature set pC, calculated from the complete blood count. The feature set pC alone achieved a COVID-19 mortality classification with an AUC value of 0.9844 on the dataset A1-Testing. However, if we had already obtained the original cytokine levels, the predicted cytokine levels could not improve the mortality classification because both the feature sets B + C and B + C + pC achieved the best classification (AUC = 0.9856).

The experimental data on the independent testing set A2 showed that integrating the predicted cytokine levels (pC) improved the mortality classification model using only complete blood counts (B) by 0.0145 in AUC. Furthermore, even the PC-based classification model outperformed that by 0.0108 in AUC using only complete blood counts.

**Fig. 8:**
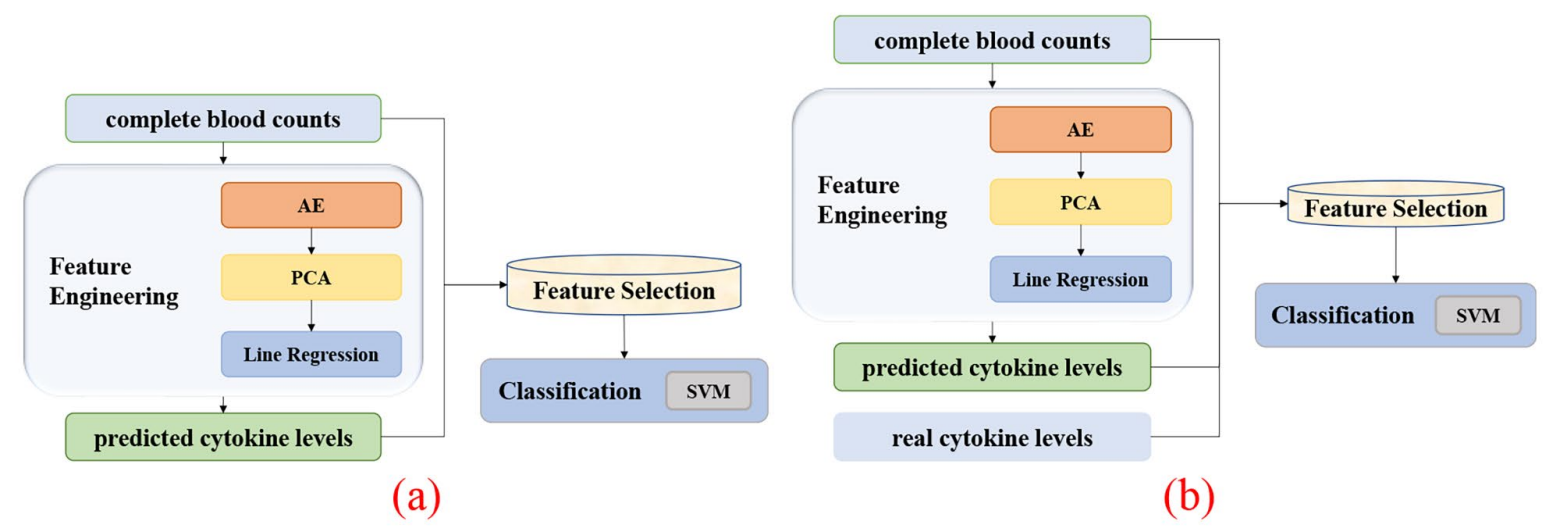
Visualizations of the experimental procedure. (**a**) Flowchart for predicting COVID-19 patients’ survival status using only the complete blood count data. (**b**) Flowchart for predicting COVID-19 patients’ survival status when the complete blood count data and the whole cytokine levels are available

The classification data suggested that complete blood counts and cytokine levels were important for the mortality risk predictions of COVID-19 patients. Even if we did not know the original cytokine levels, we could still improve the mortality classification model using the predicted cytokine levels based on the easily accessible complete blood counts (Fig. 8).

## Discussion

Complete blood counts have been widely used in clinical practice and are often performed during the hospitalization of COVID-19 patients. However, few studies have investigated the prognosis prediction of COVID-19 patients using only complete blood counts. Cytokines (IL-2R, IL-6, IL-8, TNF-α, and IL-10) were significantly associated with in-hospital mortality in COVID-19 patients [38, 39]. Therefore, hematological abnormalities such as high cytokine levels became of substantial interest to the research community as potential prognostic factors of COVID-19 deterioration [40]. For instance, the early and dramatic rise in IL-10 after SARS-CoV-2 infection may play a harmful pathological role in the severity of COVID-19 [41, 42]. Thus, this study quantitatively investigated the connections between complete blood counts and cytokine levels.

We first investigated whether the complete blood count and original cytokine level carried useful information for the COVID-19 mortality classification task. The experimental data suggested that the complete blood count and original cytokine level alone produced mortality classification AUC values of at least 0.9300. In addition, their combination improved the mortality classification AUC to values as high as 0.9878.

Then we evaluated the different numbers of PCs using the AE-filtered complete blood count for the regression estimations of the cytokine level. The experimental data showed that the predicted cytokine levels might improve the mortality classification models using only complete blood counts.

The statistical analysis also suggested that the predicted cytokine levels were more significantly associated with COVID-19 mortality than the original levels. For example, the cytokine IL-1β showed no differential representation between the deceased and recovered COVID-19 patients (*p* = 0.9701), while the model-corrected IL-1β level was significantly associated with COVID-19 mortality (*p* = 1.161e-3). Research has shown that IL-1β plays a major role in the acute inflammatory response of respiratory infections and promotes the elimination of pathogens [43, 44].

The clinical deterioration of COVID-19 patients involved multiple pathways, including chemotaxis and interleukin production [45]. Mulchandani et al. found that severe COVID-19 was characterized by significantly increased levels of pro-inflammatory cytokines (IL-6, IL-8, IL-10, IL-2R, and TNF- α) [46], which was supported by our observation that several pro-inflammatory cytokines in the dead COVID-19 patients had significantly higher levels than those in the alive patients. Lu proposed that the combined roles of IL-10 in promoting systemic inflammatory cytokine production and stimulating T-cell activation and proliferation in COVID-19 patients may contribute to a lethal immunopathological process [41]. The predicted IL-10 levels in this study showed much more significant associations with COVID-19 mortality (p-value = 3.630e-10) than the original levels (*p*-value = 8.787e-6). IL-8 was a potent neutrophil chemokine known to have a role in inflammation and host defense [47]. The IL-8 levels recovered from the complete blood counts improved the COVID-19 mortality association from the *p*-value = 3.305e-5 of the original IL-8 levels to the significance *p*-value = 3.579e-9.

Unfortunately, it is not always possible to have the cytokine levels of COVID-19 patients because of the additional detection cost of radioimmunoassay or other technologies and the availability of detection devices. However, cytokines play an important role in the prognosis of COVID-19 and other immunological diseases. Therefore, this study presented a proof-of-concept method to predict the cytokine levels based on the complete blood counts when the cytokine levels cannot be measured for the investigated patients.

Comparing the samples with (A1-Testing) and without (A2) the complete blood counts suggested that the predicted cytokine levels did not improve or worsen the COVID-19 mortality classification. However, the predicted cytokine levels could improve the mortality classification if we only had the complete blood counts. Since the complete blood counts were more economically acceptable than the other clinical diagnosis technologies like cytokine detection or medical imaging, the technology proposed in this study may be regularly used in clinical practice.

The main limitation of this study was its small sample size. This study used one of the largest publicly available COVID-19 datasets, but more external validation datasets with spatial-temporal diversities may further improve the validity and generalizability of the proposed model. In addition, many existing datasets provided fewer features than the dataset used in this study. For example, some researchers explored clinical and laboratory data commonly used in clinical practice to quickly screen COVID-19 patients [48]. Similarly, Gök and Avila used the available blood analysis data of COVID-19 patients to predict their mortality [49, 50]. Lorenzo Famiglini used complete blood counts to predict admission to the intensive care unit in the next 5 days [11]. Huyut et al. studied 2,597 samples to predict the mortality of COVID-19 patients, but they only had 12 indicators that overlapped with our study [51].

## Conclusions

This study presented a proof-of-principle investigation for predicting cytokine levels using complete blood counts and demonstrated that the COVID-19 mortality classification task could be significantly improved using these predicted cytokine levels. It is also interesting to observe that the predicted cytokine levels were much more significantly associated with COVID-19 mortality than the original ones. This suggested that the prediction model might have reduced the noise in the original cytokine levels. The exploratory investigation in this study suggested that even the test of complete blood counts alone could deliver satisfying COVID-19 mortality classification performances.

The proposed proof-of-principle model was trained using the patient samples from the beginning of the COVID-19 pandemic and might need to fit better the clinical situations in the current post-pandemic phase. However, the modeling procedure showed the predicted cytokine levels’ positive contributions to the mortality prediction of coronavirus-infected populations.

### Electronic supplementary material

Below is the link to the electronic supplementary material.

Supplementary Material 1

## Data Availability

The dataset supporting the conclusions of this article is included within the article (and its additional file). The original data was released in DOI: 10.1038/s42256-020-0180-7.

## Abbreviations

SARS-CoV-2: Severe acute respiratory syndrome coronavirus 2
GRU-D: Gated recurrent unit with trainable decay weights
IL-6: Interleukin-6
IL-10: Interleukin-10
TNF-α: Tumor necrosis factors-α
IL-8: Interleukin-8
IL-1β: Interleukin-1β
IL-2R: Interleukin-2 receptor
AE: Autoencoder
PCA: Principal component analysis
PC: Principal component
SVM: Support vector machine
AdaBoost: Adaptive boost
RF: Random forest
IFS: Incremental feature selection
NB: Bernoulli naïve bayes
GBR: Gradient boosting regressor
RFR: Random forest regressor
SVR: Support vector regression
LR: Linear regression
AUC: Area under the ROC curve
Acc: Accuracy
Sn: Sensitivity
Sp: Specificity
M.W.W: Mann-Whitney Wilcoxon
