# Supplementary material for: Reconstructing the cytokine view for the multi-view prediction of COVID-19 mortality
Source: BMC Infect Dis. 2023 Sep 21;23:622. doi: 10.1186/s12879-023-08291-z (PMC10514938; doi:10.1186/s12879-023-08291-z)
Supplement: Supplementary file 1 — Supplementary Material 1 [file 12879_2023_8291_MOESM1_ESM.docx]

# Reconstructing the Cytokine View for the Multi-view Prediction of COVID-19 Mortality

Yueying Wang^1,2,3,4^, Zhao Wang^5^, Yaqing Liu^1^, Qiong Yu^4^, Yujia Liu^5^, Changfan Luo^5^, Siyang Wang^5^, Hongmei Liu^2,3,6^, Mingyou Liu^2^, Gongyou Zhang^2^, Yusi Fan^5^, Kewei Li^1,2^, Lan Huang^1,2^, Meiyu Duan^1,3,#^, Fengfeng Zhou^1,2,3,#^.

1 College of Computer Science and Technology, Jilin University, Changchun, China 130012.

2 School of Biology and Engineering, Guizhou Medical University, Guiyang, Guizhou, China 550025.

3 Key Laboratory of Symbolic Computation and Knowledge Engineering of Ministry of Education, Jilin University, Changchun, China 130012.

4 Department of Epidemiology and Biostatistics, School of Public Health, Jilin University, Changchun, Jilin Province, China 130021.

5 College of Software, Jilin University, Changchun, China 130012.

6 Engineering Research Center of Medical Biotechnology, Guizhou Medical University, Guiyang, Guizhou, China 550025.

# Correspondence may be addressed to Fengfeng Zhou: FengfengZhou@gmail.com or ffzhou@jlu.edu.cn . Lab web site: http://www.healthinformaticslab.org/ . Phone: +86-431-8516-6024. Fax: +86-431-8516-6024. Correspondence may also be addressed to Meiyu Duan: dmy235813@163.com .

# Table S1

Baseline characteristics and risk factors associated with COVID-19 at the first test in the study population.

| **Feature** | **Survival** | **Non-survival** | ***P**** |
| --- | --- | --- | --- |
| basophil count(#) | 0.01(0.00,0.02) | 0.01(0.00,0.02) | 0.577 |
| basophil(%) | 0.20(0.00,0.30) | 0.10(0.00,0.20) | 0.001 |
| Eosinophil count | 0.01(0.00,0.04) | 0.00(0.00,0.01) | <0.001 |
| eosinophils(%) | 0.30(0.00,0.80) | 0.00(0.00,0.00) | <0.001 |
| hematocrit | 37.71±4.06 | 36.73±4.98 | 0.141 |
| hemoglobin | 128.00(122.00,140.00) | 126.50(115.00,139.00) | 0.260 |
| lymphocyte count | 1.02(0.72,1.43) | 0.51(0.37,0.73) | <0.001 |
| lymphocyte(%) | 23.60(16.00,33.10) | 6.20(3.18,10.73) | <0.001 |
| mean corpuscular hemoglobin | 30.60(29.50,31.90) | 31.10(29.90,31.78) | 0.343 |
| mean corpuscular hemoglobin concentration | 344.00(334.00,351.00) | 346.50(336.25,354.00) | 0.222 |
| mean corpuscular volume | 89.40(86.70,91.40) | 89.20(86.13,93.48) | 0.772 |
| Mean platelet volume | 10.60(10.10,11.30) | 10.90(10.40,11.60) | 0.009 |
| monocytes count | 0.38(0.29,0.51) | 0.38(0.22,0.60) | 0.663 |
| monocytes(%) | 8.53±3.37 | 5.11±3.65 | <0.001 |
| neutrophils count | 2.99(2.36,4.04) | 8.02(4.89,12.24) | <0.001 |
| neutrophils(%) | 67.30(56.60,75.50) | 87.90(82.98,94.00) | <0.001 |
| Platelet count | 199.00(159.00,248.00) | 156.00(114.25,222.00) | <0.001 |
| platelet large cell ratio | 30.26±7.08 | 33.01±7.79 | 0.010 |
| PLT distribution width | 12.00(10.90,13.90) | 12.75(11.80,14.65) | 0.011 |
| RBC distribution width SD | 39.50(37.60,41.20) | 41.50(38.85,45.10) | <0.001 |
| Red blood cell count | 4.30(3.94,4.57) | 4.09(3.71,4.54) | 0.089 |
| Red blood cell distribution width | 12.10(11.80,12.80) | 12.85(12.20,13.78) | <0.001 |
| thrombocytocrit | 0.21(0.17,0.26) | 0.17(0.13,0.24) | <0.001 |
| White blood cell count | 4.97(3.64,5.71) | 9.07(5.77,13.28) | <0.001 |

Continuous variables were summarized as mean (standard deviation) or median (quartile). T test or Mann-Whitney U test was used to compare the characteristics of the patients who survived or died based on the data distributions of the individual variables. P≤0.05 was considered as being statistically significant.
